# Three *GLI2* mutations combined potentially underlie non‐syndromic cleft lip with or without cleft palate in a Chinese pedigree

**DOI:** 10.1002/mgg3.714

**Published:** 2019-08-06

**Authors:** Peiqi Meng, Huaxiang Zhao, Wenbin Huang, Yunfan Zhang, Wenjie Zhong, Mengqi Zhang, Peizeng Jia, Zhibo Zhou, Gulibaha Maimaitili, Feng Chen, Jieni Zhang, Jiuxiang Lin

**Affiliations:** ^1^ Department of Orthodontics Peking University School and Hospital of Stomatology Beijing PR China; ^2^ Department of Oral and Maxillofacial Surgery Peking University School and Hospital of Stomatology Beijing PR China; ^3^ Department of Stomatology The Fifth Affiliated Hospital of Xinjiang Medical University Urumqi PR China

**Keywords:** cleft lip and/or palate, *GLI2*, hereditary pedigree, pathogenic mutation, whole‐exome sequencing

## Abstract

**Background:**

Nonsyndromic cleft lip with or without cleft palate (NSCL/P) is the most common craniofacial birth defect. Its etiology is complex and it has a lifelong influence on affected individuals. Despite many studies, the pathogenic gene alleles are not completely clear. Here, we recruited a Chinese NSCL/P family and explored the candidate causative variants in this pedigree.

**Methods:**

We performed whole‐exome sequencing on two patients and two unaffected subjects of this family. Variants were screened based on bioinformatics analysis to identify the potential etiological alleles. Species conservation analysis, mutation function prediction, and homology protein modeling were also performed to preliminarily evaluate the influence of the mutations.

**Results:**

We identified three rare mutations that are located on a single chromatid (c.2684C > T_p.Ala895Val, c.4350G > T_p.Gln1450His, and c.4622C > A_p.Ser1541Tyr) in *GLI2* as candidate causative variants. All of these three mutations were predicted to be deleterious, and they affect amino acids that are conserved in many species. The mutation c.2684C > T was predicted to affect the structure of the GLI2 protein.

**Conclusion:**

Our results further demonstrate that *GLI2* variants play a role in the pathogenesis of NSCL/P, and the three rare missense mutations combined are probably the potential disease‐causing variants in this family.

## 1 INTRODUCTION

Cleft lip with or without cleft palate is a common birth defect found in more than 300 recognizable syndromes, but more often it is observed as an isolated birth defect (~70%) (Calzolari et al., 2007) called non‐syndromic cleft lip with or without cleft palate (NSCL/P). NSCL/P has been reported to affect 1 in 500 to 1 in 2,500 newborns depending on geographical origins and ethnic groups. In general, East Asian and Native American populations have the highest rates, followed by Caucasians, with African populations exhibiting the lowest rates (Stanier & Moore, 2004). Since the defect causes not only significant facial anomalies but also speech and feeding difficulties, malnutrition, infections, and psychiatric effects, and requires complex, multi‐disciplinary interventions including surgery, orthodontic dentistry, speech therapy, and psychological treatment, it has lifelong implications for affected individuals and imposes a substantial burden on families and society (Wehby & Cassell, 2010).

NSCL/P is an etiologically heterogeneous birth defect, where both genetic and environmental factors contribute to disease risk (Dixon, Marazita, Beaty, & Murray, 2011). With the development of genomic technologies, the utilization of genome‐wide association studies (GWAS), candidate genes, and animal models have led to a deeper insight into the etiology of NSCL/P (Dixon et al., 2011). Especially GWAS, which have been widely applied in recent years, have identified quite a few novel loci that are significantly associated with NSCL/P (Beaty et al., 2010; Birnbaum et al., 2009; Ludwig et al., 2012; Mangold et al., 2010; Sun et al., 2015; Yu et al., 2017). However, GWAS usually investigate variants with high allele frequencies (usually > 0.05) (Birnbaum et al., 2009), whereas some variants that are rare in the population are also pathogenic for NSCL/P, leading to difficulties in determining the association between NSCL/P and variants with low allele frequencies (Vieira, 2008). Furthermore, it has been estimated that only about 25%–30% of the variance in risk for NSCL/P can be attributed to the common variants identified by GWAS (Ludwig et al., 2017). In addition, a recent large‐scale genome‐wide analysis has shown that 26 identified loci collectively account for 10.94% of the heritability for NSCL/P in the Chinese population (Yu et al., 2017). Together, these analyses suggest that the genetic factors accounting for NSCL/P have not been fully characterized, especially in the Chinese population.

GLI2 is a member of the GLI family zinc finger proteins, which are the only known transcriptional effectors of the sonic hedgehog (SHH) signaling pathway, one of the key regulatory networks involved in animal development that is conserved from flies to humans (Jiang & Hui, 2008). The GLI2 protein is vital for embryogenesis (Ding et al., 1998; Mo et al., 1997; Motoyama et al., 1998). A great many of its target genes are regulators of cell determination, specification, proliferation, and death, as well as of stem cell renewal and differentiation. Misregulation of the *GLI2* (OMIM: 165230) gene could therefore lead to unfavorable developmental and pathological consequences (Hui & Angers, 2011). Mutations in *GLI2* have been identified in patients with orofacial cleft (Bertolacini, Ribeirobicudo, Petrin, Richiericosta, & Murray, 2012; Mo et al., 1997; Simioni, Araujo, Monlleo, Maurer‐Morelli, & Gil‐Da‐Silva‐Lopes, 2015; Vieira et al., 2005), holoprosencephaly (Bear et al., 2014; Kevelam et al., 2012), and pituitary anomalies (Roessler et al., 2003, 2005). Some variants of *GLI2* have been detected in patients who present with cleft lip/palate by Sanger sequencing and are not in unrelated controls without orofacial cleft (Simioni et al., 2015). In a case report, a holoprosencephaly patient with bilateral cleft lip and palate was found to carry a submicroscopic heterozygous deletion of the *GLI2* gene (Kevelam et al., 2012). In addition, mutant mice deficient for *GLI2* exhibit severe skeletal abnormalities, including cleft palate (Mo et al., 1997).

Here, we used whole exome sequencing (WES) to identify the underlying potential cause of NSCL/P in a Chinese pedigree with hereditary NSCL/P to further expand the genetic pathogenic spectrum and our knowledge of the genetic mechanisms of NSCL/P.

## 2 MATERIALS AND METHODS

### 2.1 Ethical compliance

This study was approved by the Ethics Committee of the Peking University School and Hospital of Stomatology (PKUSSIRB‐201520012) and was conducted according to the Declaration of Helsinki principles. Understanding and written informed consent was obtained from the subjects themselves or from their guardians.

### 2.2 Human subjects

A Hui Chinese family from Urumqi, Xinjiang with NSCL/P of unknown etiology was recruited at Peking University School and Hospital of Stomatology. We also recruited an independent cohort of 31 NSCL/P‐unaffected subjects to verify the allele frequency. All of the subjects were interviewed and clinically assessed by at least two experienced oral and maxillofacial surgeons. Full clinical checkups were completed to identify any further anomalies such as congenital heart disease, cyclopism, polydactyly, and so on, which would suggest an underlying syndrome. Additional demographic information was obtained through a detailed questionnaire, including gender, age, nationality, and maternal exposure history of smoking, drinking, medicine, radiation, poisons, and chemicals during the first trimester of pregnancy.

We collected 2–4 ml peripheral blood samples from each accessible participant. The buccal swabs from the maternal grandparents of the proband were carefully acquired and reserved in TIANGEN Sample Preservation Buffer (Tiangen, Beijing, China). Genomic DNA was extracted using the QIAamp DNA Blood Mini Kit (Qiagen, Hilden, Germany) or TIANGEN Hi‐Swab DNA Kit (Tiangen, Beijing, China) according to the manufacturer's instructions. The quality and quantity of the DNA samples were assessed using a NanoDrop 8000 (Thermo Scientific, Waltham, MA) and agarose gel electrophoresis.

### 2.3 Whole exome sequencing

DNA libraries with enrichment of exonic sequences were constructed for the proband, as well as the elder sister and parents of the proband. High‐throughput sequencing of the libraries was performed, and raw sequencing reads were generated using the BGISEQ‐500 platform (BGI Inc., Beijing, China) (Zhao, Zhang, et al., 2018; Zhao, Zhong, et al., 2018). Adapter sequences, low‐quality sequences, and undetected bases were excluded. The remaining reads for each sample were mapped to the human reference genome (GRCh37/hg19) using Burrows‐Wheeler Aligner software. Variant discovery analysis was performed using the Genome Analysis Toolkit (GATK) according to the guidelines provided (https://software.broadinstitute.org/gatk/). Repeat reads were removed using Picard Tools (http://broadinstitute.github.io/picard/). Local realignment and base quality recalibration were conducted using the GATK. Sequencing depth, target coverage, and capture specificity were calculated based on the alignment. Subsequently, single nucleotide polymorphisms (SNPs) and insertions/deletions (InDels) were detected and screened using HaplotypeCaller (GATK v3.3.0). SnpEff (http://snpeff.sourceforge.net/SnpEff_manual.html) was then used to perform annotation and prediction.

### 2.4 Screening of candidate causal variants

After annotation, a screening process was performed to identify the candidate gene mutations. First, we excluded variants with a minor allele frequency (MAF) ≥ 0.5% in the 1000 Genome Project database (http://www.1000genomes.org/) as well as variants in non‐coding DNA sequences, including the 3'‐UTR, 5'‐UTR, downstream, upstream, intergenic, intron, synonymous, stop‐retained, and noncoding exon variants. Next, a Mendelian inheritance model was used to narrow down the candidates. Only heterozygous variants present in all individuals with cleft and not present in any individuals without cleft were retained. Then, the MAF of each retained variant was verified in several public databases by handwork, including Ensembl (http://asia.ensembl.org/index.html), ExAC (http://exac.broadinstitute.org), and NCBI dbSNP (https://www.ncbi.nlm.nih.gov/snp/), and those with high frequency were screened out. In addition, since we used the allele positions to select variants that accord with the dominant inheritance model, we checked and excluded variants with the same allele positions but with inconsistent variant forms in subjects D1 and D2, such as c.859A > G (NM_001146181.2) in D1 and c.859A > AGC (NM_001146181.2) in D2. Finally, the literature was reviewed for candidate variants with potential significant relationships to the phenotype and possible deleterious effects on craniofacial structures.

### 2.5 Confirmation by Sanger sequencing

We performed Sanger sequencing for validation of three potential causal variants on the family members. For the novel *GLI2* (NM_005270.4) mutation we identified, which has not been reported in the databases, we further performed Sanger sequencing for validation of the MAF on 31 unaffected Chinese subjects. Polymerase chain reaction (PCR) primers were designed (forward: 5′‐AAGCAGCCACCCACCCTTGT‐3′; reverse: 5′‐AATGTCAGCCGTGAAGAAGC‐3′) using Primer Premier 5.0. The reaction mixture in a 50‐μl volume was made using 0.5 μl *TaKaRa LA Taq* (5 U/μl), 25 μl 2 × GC Buffer I, 8 μl dNTP Mixture (2.5 mM each), 1 μl DNA template (80 ng/μl), 1 μl forward primer (10 mM), 1 μl reverse primer (10 mM), and 13.5 μl ddH2O. Since the PCR target sequence is GC‐rich, we applied hot‐start PCR and touch‐down PCR. The reaction procedures were as follows: 95°C for 15 min, the first 15 cycles of 95°C for 1 min, 70–56°C for 30 s, and 72°C for 3 min, and the last 16 cycles of 95°C for 1 min, 58°C for 30 s, and 72°C for 3 min. The Sanger sequencing data were analyzed using Chromas v1.0.0.1 software.

### 2.6 Cloning of the PCR target sequence

The PCR procedure was repeated for DNA samples of subjects D1 and D2, as well as the father of D2. PCR products were purified by gel extraction using an AxyPrep DNA Gel Extraction Kit (Axygen, CA) after agarose gel electrophoresis. Then, the 2,831‐bp DNA target sequence was ligated to a TOPO TA vector (Mei5, Beijing, PRC) for 1 hr at room temperature. Next, the products were transformed into *Escherichia coli* competent cells. The bacteria were plated on ampicillin‐containing Luria‐Bertani (LB) solid medium. After incubating at 37°C for 14 hr, monoclonal colonies were selected. Sanger sequencing was performed on the cloned DNA sequences to identify the arrangement of the alleles of the three variants on the chromatid.

### 2.7 Conservation analysis, functional prediction, and homology modeling

Evolutionary conservative analysis of the mutation positions was conducted using UniProt (http://www.uniprot.org/) and ClustalX V2.1. To predict the potential functional effects of the identified variants, in silico tools such as SIFT (Ng & Henikoff, 2003) (http://sift.jcvi.org/), PROVEAN (Choi & Chan, 2015) (http://provean.jcvi.org/index.php), PolyPhen‐2 (Adzhubei et al., 2010) (http://genetics.bwh.harvard.edu/pph2/), and MutationAssessor (Reva, Antipin, & Sander, 2011) (http://mutationassessor.org/r3/) were applied. Additionally, the website‐based domain prediction tool SMART (Simple Modular Architecture Research Tool) (http://smart.embl-heidelberg.de) was used to predict domains for GLI2 (Letunic & Bork, 2018; Letunic, Doerks, & Bork, 2015).

To obtain more insight into the effects of the mutations on the molecular structure of GLI2, we created a homology model. The sequence of GLI2 was downloaded from NCBI (www.ncbi.nlm.nih.gov/protein). SWISS‐MODEL (https://www.swissmodel.expasy.org/) was used for the molecular simulation (Biasini et al., 2014). Template 5ijm.1.A was used to create the model for residues 590–1116, which covers mutation site M1. Unfortunately, there are no templates coving the mutation sites M2 or M3 in the SWISS‐MODEL database. PyMOL software was used for visualization.

## 3 RESULTS

### 3.1 Phenotypic descriptions of the NSCL/P pedigree

**Figure 1:**
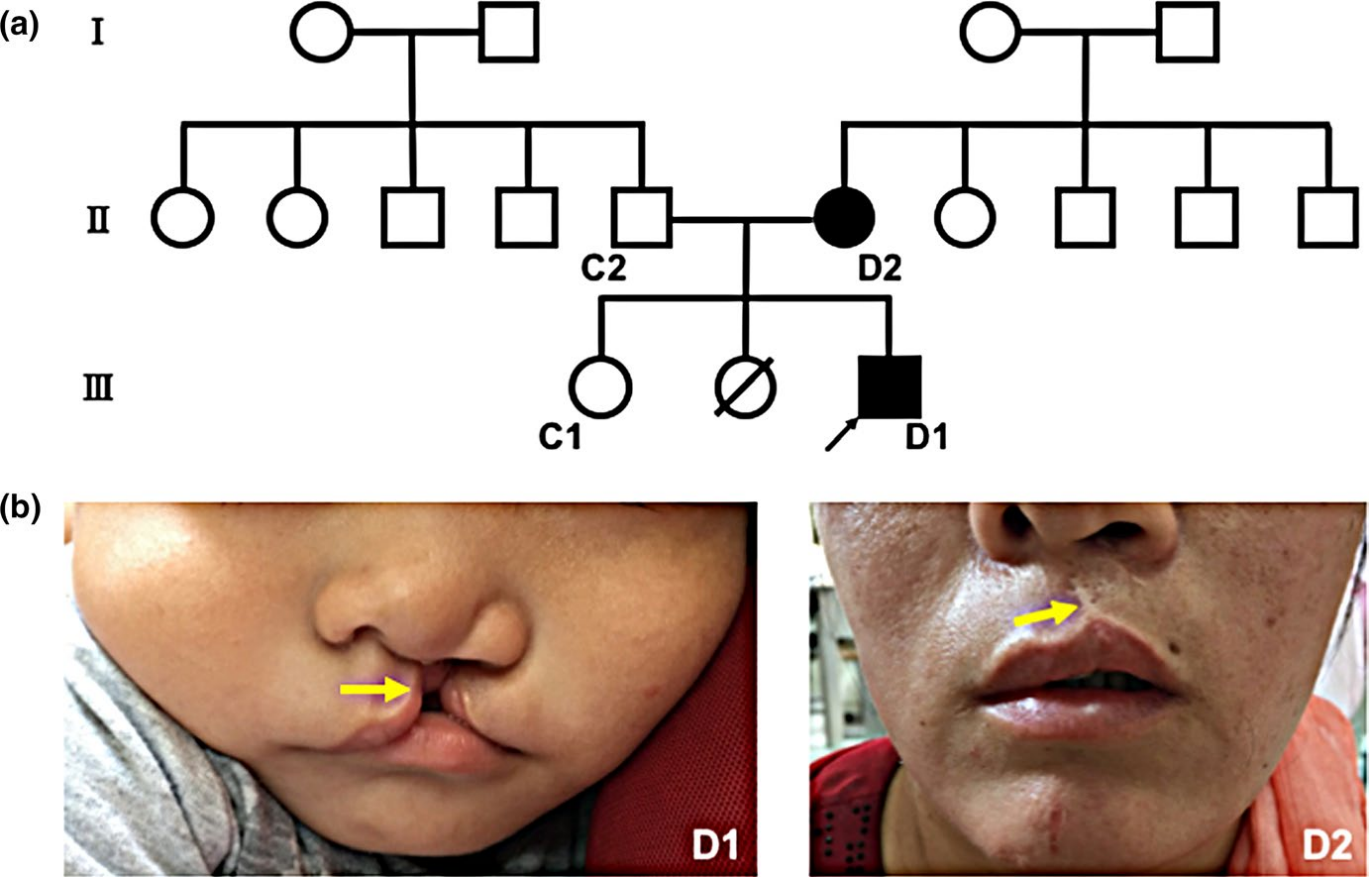
(a) Pedigree diagram of a Chinese family with NSCL/P. Filled symbols indicate the patients, while blank symbols indicate unaffected members. The black arrow indicates the proband (D1). The symbol with an oblique line indicates early death. (b) Photographs of D1 and D2 presenting their phenotypes. Unfortunately, photographs showing their palates were not accessible. The yellow arrow of subject D2 indicates the scar from left cleft lip repair

We recruited a three‐generation Hui Chinese family with NSCL/P (Figure 1a). The male proband (D1) presented with left cleft lip and cleft palate, while his mother (D2) exhibited left cleft lip only (Figure 1b). Both patients received a detailed general physical examination, including eyes and vision, external ear morphology and hearing, cranio‐skeletal development, long bone morphology, neuromuscular and motor system function, cardiovascular system function, and external genital system morphology, to eliminate the possibility of systemic malformation. Therefore, these two patients were diagnosed with NSCL/P.

The father (C2) and two elder sisters of the proband D1 were not affected with cleft lip/palate. One of the elder sisters died from unknown causes in infancy. The grandfather, grandmother, as well as uncles, and aunts from both the paternal and maternal sides to whom we had no access for physical examinations claimed to be not affected with cleft lip/palate through telephone. The photographs of the father and mother of D2 taken by themselves and mailed to us were presented in Supporting Information Figure S1a.

During the pregnancy, D2 was not exposed to diseases, smoking, alcohol, radioactivity, or chemical teratogens, and there was no supplementation with folic acid, iron, or vitamin B6.

### 3.2 Three rare *GLI2* mutations identified by WES and variation screening

We performed WES on the two patients (D1 and D2) as well as two unaffected family members (C1 and C2) in this NSCL/P hereditary family. These samples yielded 21.55 Gb data of mappable targeted exome sequences on average, with a mean sequencing depth of 229.03‐fold. In total, 97.63% of the exonic regions were covered at least 20‐fold, indicating high sequencing quality (Table 1).

**Table 1:** Summary of whole exome sequencing data and alignment

Sample	Total effective yield (Gb)	Average sequencing depth	Q20%	Q30%	Mapping rate on genome (%)	Coverage of exome (%)	Target capture specificity (%)	Fraction of target covered ≥ 4x (%)	Fraction of target covered ≥ 20x (%)
D1	23.62	227.68	97.93	92.13	99.95	99.91	56.85	99.59	97.53
D2	24.65	268.58	97.90	92.02	99.97	99.73	64.25	99.41	97.82
C1	19.23	211.10	97.37	90.05	99.95	99.72	64.73	99.38	97.42
C2	18.69	208.74	97.69	91.01	99.95	99.87	65.88	99.58	97.74
Average	21.55	229.03	97.72	91.30	99.96	99.81	62.93	99.49	97.63

After mapping the sequences to the human GRCh37/hg19 reference genome, we identified 124,813 single nucleotide variants and indels in D1, 124,365 in D2, 118,331 in C1, and 119,723 in C2. On average, there were 68,348 heterozygous variants and 53,460 homozygous variants (Table 2). Both the quantity and quality of the sequencing met the requirements for further analysis.

**Table 2:** Summary statistics for identified variants

Sample	Total variation	Heterozygotes	Homozygotes	Exonic	Intronic	Intergenic	Splicing	Synonymous	Missense	Stop‐gain	Stop‐loss
D1	124,813	69,300	55,513	13,516	84,918	3,257	2,195	11,170	10,360	83	37
D2	124,365	70,458	53,907	13,424	84,648	3,099	2,203	11,257	10,283	76	37
C1	118,331	66,291	52,040	13,511	79,203	3,217	2,197	11,177	10,378	88	36
C2	119,723	67,343	52,380	13,647	80,210	3,145	2,233	11,128	10,467	85	35
Average	121,808	68,348	53,460	13,525	82,245	3,180	2,207	11,183	10,372	83	36

**Figure 2:**
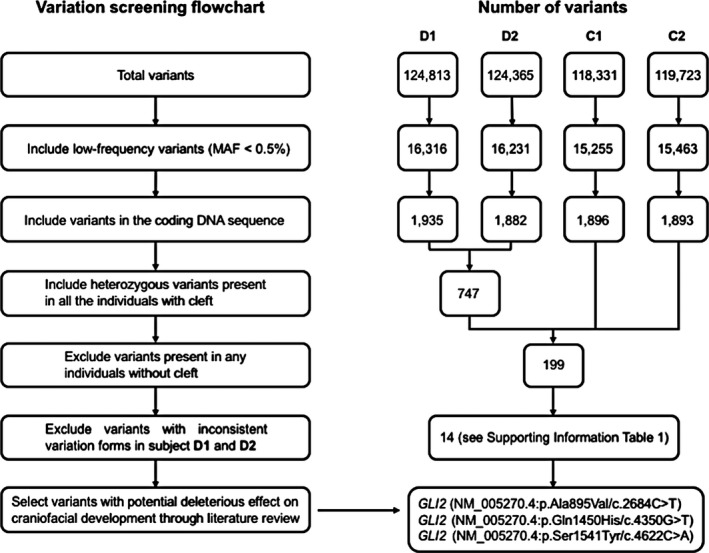
Flowchart outlining the screening process of the causative variants (left) and the number of remaining variants after each corresponding step (right)

Subsequently, we applied a screening process to identify the candidate variants (Figure 2). Variants with MAF ≥ 0.5% in the 1,000 Genomes database were filtered out, leaving 16,316, 16,231, 15,255, and 15,463 variants in subjects D1, D2, C1, and C2, respectively. Next, we focused on variants that were more likely to be pathogenic, such as nonsynonymous variants, splice acceptor‐site or donor‐site mutations, as well as frameshift insertions or deletions, resulting in 1,935, 1,882, 1,896, and 1,893 variants in D1, D2, C1, and C2 separately. Given that the family probably showed a dominant inheritance model, we selected heterozygous variants shared by D1 and D2, and 747 variants were found. In addition, variants that were present in healthy individuals C1 or C2 were excluded, leading to 199 variants left. Then, we excluded variants with MAF ≥ 0.5% in Ensembl, ExAC, and NCBI dbSNP databases or with inconsistent variation forms in subject D1 and D2, and only 14 variants were retained (Supporting Information Table S1). Finally, we reviewed previously reported genes with known roles in craniofacial development or the pathogenesis of cleft lip/palate. Eventually we identified three missense mutations M1: c.2684C > T, M2: c.4350G > T, and M3: c.4622C > A in *GLI2* (NM_005270.4), predicting p.Ala895Val, p.Gln1450His, and p.Ser1541Tyr respectively as candidate pathogenic factors in the family. Among the three mutations, M1: c.2684C > T (NM_005270.4) is novel and has not been reported in any database previously, while M2: c.4350G > T (NM_005270.4) and M3: c.4622C > A (NM_005270.4) have already been reported to be very rare with MAFs of 0.4‰ and 0.6‰ in the 1,000 Genomes database, respectively (Table 3).

**Table 3:** Allele frequencies of the three mutations in various populations reported in different databases and in our Chinese cohort

Variant No.	Coding sequence position of the variant^a^	Amino acid substitution^b^	Type	1,000 genomes all	1,000 genomes Asian populations	1,000 genomes American populations	1,000 genomes European populations	ExAC	Our cohort of 31 unaffected Han Chinese subjects
M1	c.2684C > T	p.Ala895Val	missense	/	/	/	/	/	0
M2	c.4350G > T	p.Gln1450His	missense	0.00040	0.00200	0	0	0.00147	NP
M3	c.4622C > A	p.Ser1541Tyr	missense	0.00060	0.00300	0	0	/	NP

a The coding sequence position within the GLI2 transcript, NM_005270.4.

b Amino acid substitution is for the GLI2 protein, NP_005261.2.

### 3.3 Verification of the *GLI2* mutations by Sanger sequencing

**Figure 3:**
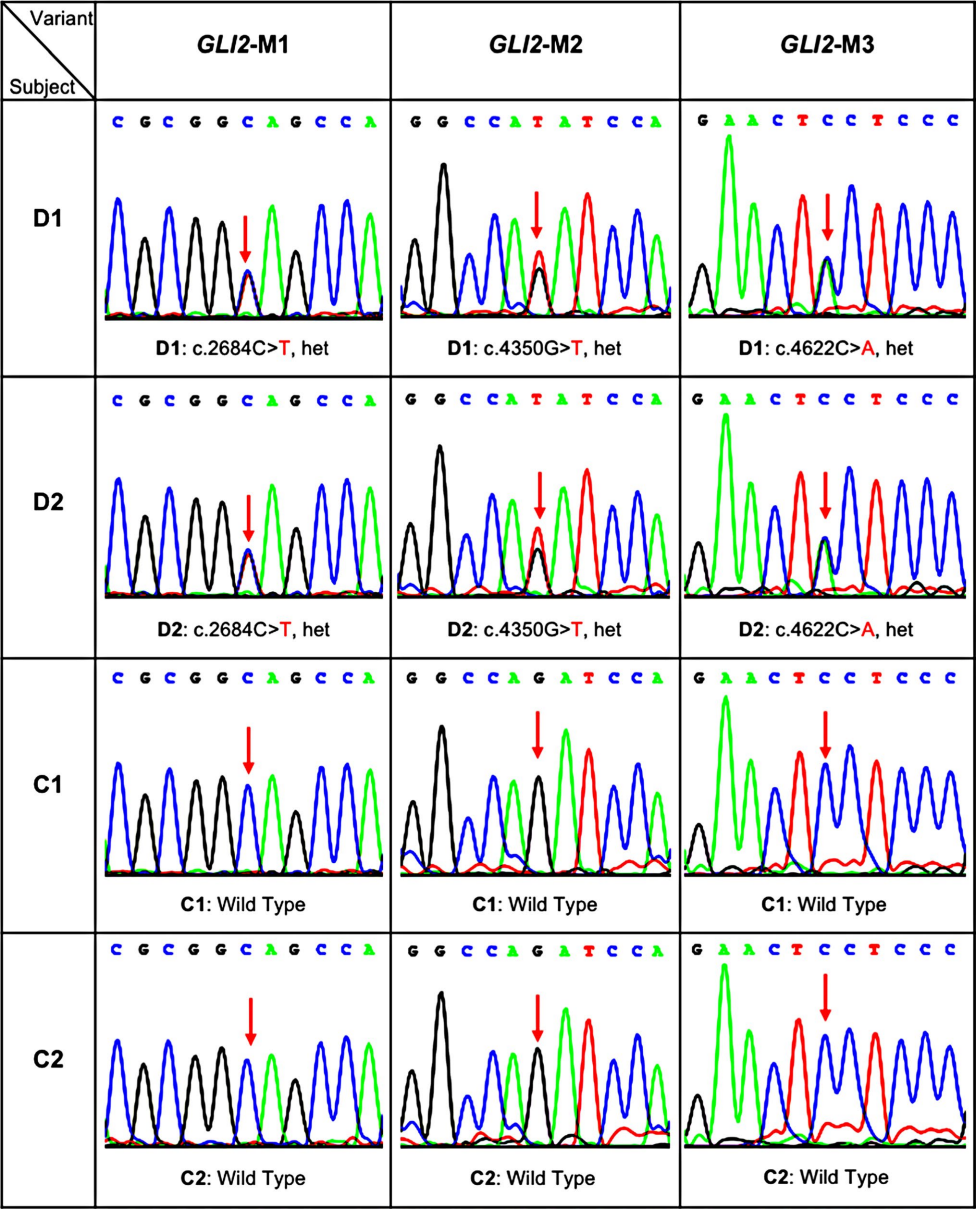
Sanger sequencing validations of the three potential causative mutations in subjects D1, D2, C1, and C2 of the family. All three mutations in D1 and D2 are heterozygous, while C1 and C2 are the wild type. Red arrows indicate the positions of the causative mutations

Sanger sequencing performed on the independent healthy Chinese cohort did not detect any c.2684C > T variant (Table 3, Supporting Information Figure S2), roughly excluding the possibility of high frequency for the mutation M1. We also validated the three *GLI2* mutations in both patients (D1 and D2) and unaffected subjects (C1 and C2) of the family by Sanger sequencing (Figure 3). Both patients were heterozygous for all of the three mutant alleles, while C1 and C2 had the wild‐type (WT) alleles, which agreed with the WES results and the autosomal dominant inheritance pattern. In addition, the father of the subject D2 was heterozygous for all of the three mutant alleles, while the mother of the subject D2 had the wild‐type alleles (Supporting Information Figure S1b).

### 3.4 Identification of the allele arrangements on the chromatid by DNA cloning

**Figure 4:**
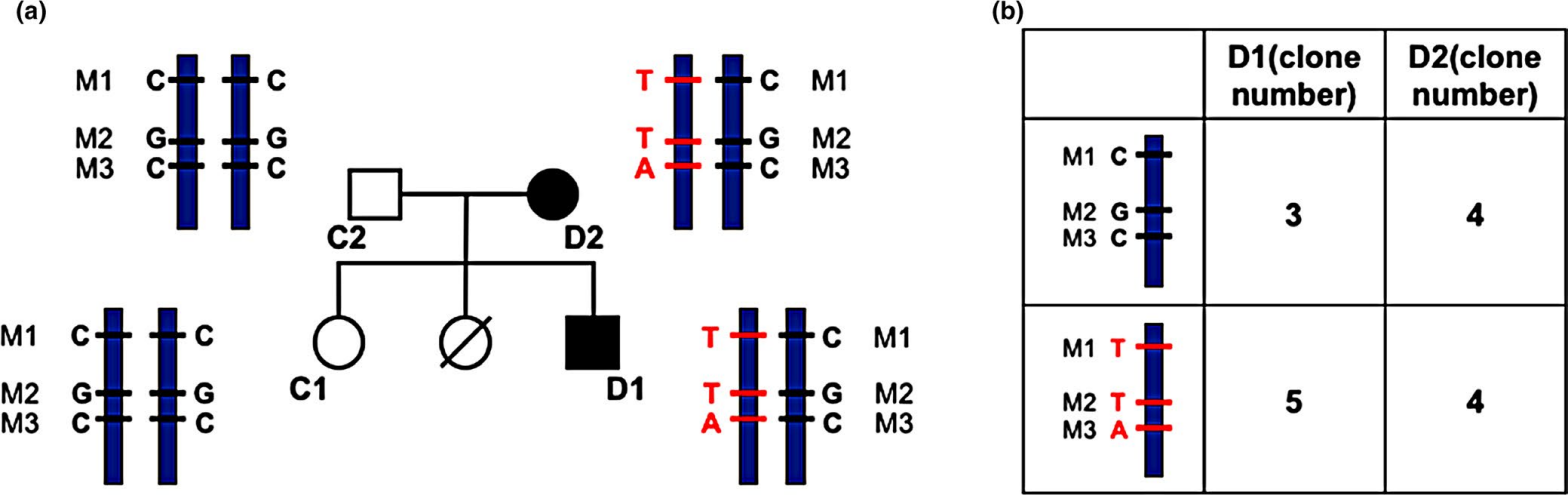
(a) Schematic indicating the arrangements of the three *GLI2* missense mutations on the two chromatids of the homologous chromosome in subjects D1, D2, C1, and C2. The short horizontal lines indicate the sites of the alleles. The red lines and the alleles shown in red font indicate the mutant, while the black lines and the alleles shown in black font indicate the wild type. (b) Table showing the number of clones for each type of allele arrangement on the chromatid in D1 and D2. Since the alleles are complementary on each site, both of the two types of chromatid support the hypothesis that the three mutant alleles are located on a single chromatid

To identify whether these three *GLI2* mutations are located on the same chromatid, we cloned the DNA sequences containing the three loci in subjects D1, D2, and the father of D2, all of whom have heterozygous genotypes for all three loci. Among eight monoclonal colonies selected for D1, three clones exhibited WT alleles in all of the three loci (M1: C, M2: G, M3: C) and five clones showed mutant alleles in all of the three loci (M1: T, M2: T, M3: A), a consistent result indicating that in the genome of patient D1, the three mutations of *GLI2* are located on a single chromatid. In D2, there were four clones with WT alleles in all three loci (M1: C, M2: G, M3: C) and four clones with mutant alleles in all three loci (M1: T, M2: T, M3: A). This indicates that in the genome of patient D2, the three *GLI2* mutations are also located on a single chromatid. In the father of D2, five clones presented WT alleles in all three loci (M1: C, M2: G, M3: C) and three clones showed mutant alleles in all three loci (M1: T, M2: T, M3: A), indicating that the three mutations of *GLI2* are also located on a single chromatid in the genome of the father of D2. It suggests that all of the three mutant alleles of the *GLI2* gene were delivered from the father of the subject D2 to the subject D2, and then passed down to the subject D1. (Figure 4a,b, Supporting Information Figure S3).

### 3.5 Potential deleterious effects of the three *GLI2* mutations

**Figure 5:**
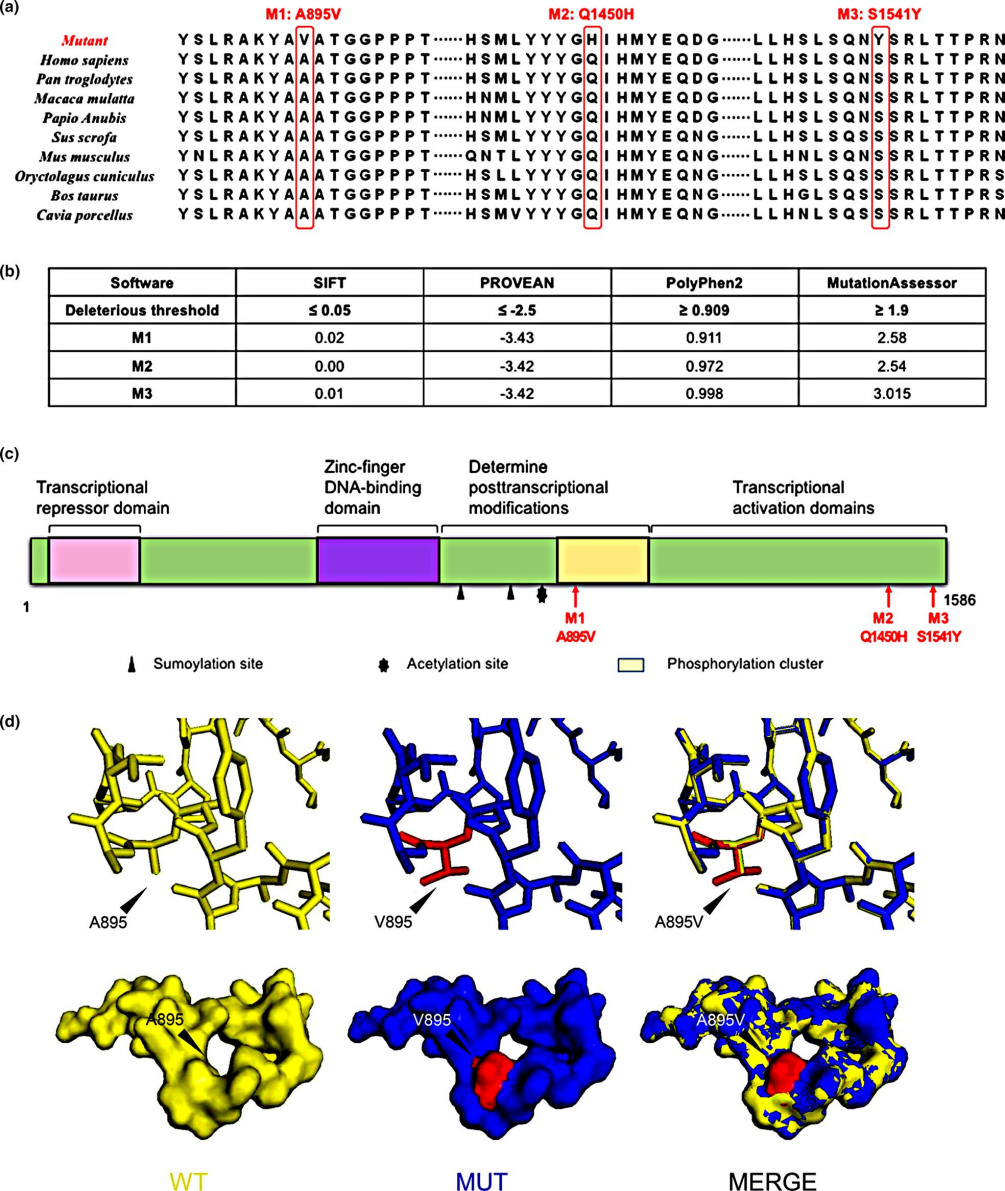
(a) Multiple sequence alignment showing evolutionary conservation of the three residues in GLI2. All of the three amino acids are highly conserved among vertebrates. (b) The predicted impact scores of the three mutations using four in silico functional prediction tools, SIFT, PROVEAN, PolyPhen‐2, and MutationAssessor. (c) Schematic representation of domains in the GLI2 protein. Various protein domains and modification sites of GLI2 are depicted. Red arrows indicate the sites of the three mutations found in this study. (d) Homology models of the wild type (WT) (yellow), mutant (MUT) (blue), and merged. The differences between the WT and the MUT are shown in red

A multiple sequence alignment of GLI2 showed that the residues Ala895, Gln1450, and Ser1541 are evolutionarily conserved in all of the species compared, including *Homo sapiens*, *Pan troglodytes*, *Macaca mulatta*, *Papio Anubis*, *Sus scrofa*, *Mus musculus*, *Oryctolagus cuniculus*, *Bos Taurus*, and *Cavia porcellus* (Figure 5a).

Moreover, several computational algorithms predicted that all three mutations could cause disease. The SIFT scores (deleterious score ≤ 0.05) were 0.02, 0.00, and 0.01 for the mutations M1 (c.2684C > T_p.Ala895Val), M2 (c.4350G > T_p.Gln1450His), and M3 (c.4622C > A_p.Ser1541Tyr), respectively. The PROVEAN scores (deleterious score ≤ −2.5) were −3.43, −3.42, and −3.42 for the mutations M1, M2, and M3, respectively. The PolyPhen‐2 scores (deleterious score ≥ 0.909) were 0.911, 0.972, and 0.998 for the mutations M1, M2, and M3, respectively. The MutationAssessor scores (deleterious score ≥ 1.9) were 2.58, 2.54, and 3.015 for the mutations M1, M2, and M3, respectively (Figure 5b). All of the three missense mutations were predicted to be potentially deleterious.

In the GLI2 protein, the mutation M1 (p.Ala895Val) affects an amino acid located in the phosphorylation cluster and around acetylation sites and sumoylation sites, which are located in the C‐terminal to DNA‐binding domain that comprises five tandem C2H2 zinc‐fingers. The mutations M2 (p.Gln1450His) and M3 (p.Ser1541Tyr) lie in the C‐terminal activation domain of the GLI2 protein (Figure 5c). This further suggests that the three mutations of *GLI2* are likely to impact the function of the protein.

Moreover, a homology model for GLI2 revealed differences in the structures between the WT and the mutant‐M1 proteins (Figure 5d). By changing alanine 895 to valine, the hydrophobicity distribution differed between the mutant‐M1 and the WT proteins at C‐terminal. The surface hydrophobicity was increased and wider in the mutant‐M1 model compared to the WT model. The effect of the Ala895Val substitution on the local structure of GLI2 may affect the protein's phosphorylation, which plays an important role in its transformation from the full‐length to active form or repressor form.

## 4 DISCUSSION

In this study, we performed WES in a Chinese family with inherited NSCL/P. After screening and Sanger sequencing, three missense mutations in the *GLI2* gene were identified as possibly etiological variants for the family. Among the three mutations, c.2684C > T is novel, while c.4350G > T and c.4622C > A have been deposited in databases (1,000 Genomes, Ensembl, ExAC, and NCBI dbSNP) and have already been reported to be rare variants. We demonstrated that the three mutant alleles are located on a single chromatid, giving rise to a mutant protein with replaced amino acids in all three sites. The three sites, with valine (Val), histidine (His), and tyrosine (Tyr) replacing alanine (Ala), glutamine (Gln), and serine (Ser) at the 895th, 1450th, and 1541th residues, respectively, are all conserved in a multiple sequence alignment of multiple species. In addition, homology models and in silico predictions indicated that these mutations may alter the normal structure and function of the protein and are therefore potential causes of the phenotype in this family.

As a genetically complex disease, NSCL/P may have multiple genetic models. We considered all of the possible modes of inheritance for this family, including dominant, recessive, and co‐dominant models. Based on the family pedigree information in this study, an autosomal dominant Mendelian inheritance model was considered the best fit, because in this family, only the mother (D2) and the son (D1) are affected with NSCL/P, while the others are not. We firstly inferred that the three *GLI2* mutations were de novo in patient D2. However, when we tried our best to get DNA samples of the parents of D2, we found that the father of D2, who had claimed to be not affected with cleft, had mutant alleles in all of the three loci of the *GLI2* gene. It suggested that the three mutations were delivered from the father of D2 to the subject D2, and then to the subject D1, and the effects of the three mutations might be incompletely penetrant, as reported in some previous genetic studies of clefting (Basha et al., 2018; Cox et al., 2018; Eshete et al., 2018). Besides, we noticed that the phenotypes of the two NSCL/P affected patients are not fully identical in this family. The mother (D2) manifests cleft lip only, while the son (D1) presents cleft lip with cleft palate, exhibiting variable expressivity. We had considered that there might be specific variants contributing to the phenotype of cleft palate in the son. Nevertheless, after screening, we did not find any cleft‐related rare variants that were present in D1 and not present in subjects D2, C1, or C2 (data not shown).

The reasons leading to reduced penetrance and variable expressivity could be various. One of the possibilities is subclinical phenotypes of the lip and palate (Dixon et al., 2011; Leslie, 2012), including microform clefts (also known as congenital healed cleft lip), defects of the *orbicularis oris* muscle (Neiswanger et al., 2007; Weinberg et al., 2008), bifid uvula, and submucous cleft palates. Other associated subclinical features which could also be present in “unaffected” relatives include craniofacial measures (Weinberg, Maher, & Marazita, 2006), dental anomalies (tooth agenesis, microdontia, and supernumerary teeth) (Vieira, Mchenry, Daackhirsch, Murray, & Marazita, 2008), brain structural differences (Nopoulos, Richman, Murray, & Canady, 2002), and lip prints (Scott et al., 2005). Another commonly used hypothesis to explain phenotypic variability is genetic modifiers, which could affect the phenotypic expression of the other genes. The existence of modifier genes has already been demonstrated in mouse models, as it was common to find phenotypic differences between different inbred strains (Cormier et al., 1997; MacPhee et al., 1995). Recently, it was also illustrated with an example in which an individual is heterozygous for both a regulatory variant and a pathogenic coding variant. The two possible haplotype configurations would result in either decreased penetrance of the coding variant, if it was on the lower‐expressed haplotype, or increased penetrance of the coding variant, if it was on the higher‐expressed haplotype (Castel et al., 2018). Alternatively, affected individuals with variable expressivity and unaffected individuals in this pedigree may share the same genetic risk factors but be differentially exposed to environmental factors, such as maternal nutritional deficiency during the periconceptional period, leading to clefts in the presence of risk alleles (Houston, 2012).

Our study identified three missense mutations in the *GLI2* coding sequence of the NSCL/P affected mother and son, which are possibly disease‐causing variants for the family. Consistent with this, there is evidence from animal models demonstrating a critical role for *GLI2* in skeletal development. *Gli2* mutant mice exhibit severe skeletal abnormalities, including cleft palate (Mo et al., 1997). In a sequence screening of NSCL/P patients, the variants p.Ser1555Pro and p.Ala268Val of GLI2 were found in patients presenting with primary features of cleft lip (Bertolacini et al., 2012). Vieira et al. performed direct sequencing of 184 cases with NSCL/P and linkage disequilibrium studies in 501 family triads affected with cleft, identifying p.Ser1213Tyr in GLI2 as potentially etiologic for NSCL/P (Vieira et al., 2005). Moreover, variants of *GLI2* have already been shown to play a pivotal role in the etiology of holoprosencephaly or holoprosencephaly‐like phenotype, of which one of the characteristic facial features are cleft lip/palate (Bear et al., 2014).

In vertebrates, GLI2 is a dedicated transcription factor involved in intracellular signal transduction regulated by the SHH signaling pathway. GLI2 protein includes a DNA‐binding domain comprising five C2H2 zinc‐fingers, an N‐terminal transcriptional repressor domain, and a C‐terminal activation domain (Briscoe & Thérond, 2013). In addition, a region C‐terminal to the DNA‐binding domain contains clusters of phosphorylation sites that are essential for the proteolytic processing that removes the C‐terminal region of the protein to yield a transcriptional repressor (GLI2R).

In the absence of a signal, GLI2 is sequentially phosphorylated by protein kinase A, casein kinase I, and glycogen synthase kinase 3β, which promotes the ubiquitylation of GLI2. This leads to the proteolytic cleavage, and the C‐terminal activation domain is removed to generate the repressor form (GLI2R). The truncated protein, which retains its DNA‐binding domain, then translocates to the nucleus to repress target gene expression (Hui & Angers, 2011). In the presence of SHH ligand, the binding of SHH to a receptor complex that consists of PTCH1 (another gene known to be involved in NSCL/P) releases the inhibition of Smoothened (SMO) (Ayers & Thérond, 2010). The activation of SMO promotes the formation of the transcriptional activator (GLI2A) by blocking GLI2 proteolytic processing. In addition, further posttranslational modifications, including phosphorylation, acetylation, and sumoylation, also contribute to the generation of GLI2A (Cox, Briscoe, & Ulloa, 2010). Then, GLI2A translocates to the nucleus and replaces GLI2R on target genes to activate transcription. Since one of the variants identified in this study, M1 (Ala895Val), is located in the phosphorylation cluster near the acetylation sites and sumoylation sites, it is likely to interfere with the posttranslational modification, which plays a critical role in the activation of GLI2. The other two variants, M2 (Gln1450His) and M3 (Ser1541Tyr), are in the C‐terminal activation domain. Thus, M2 and M3 mutations could disturb the structure and the function of the transcriptional activator GLI2A. Given that all three mutations might have a negative effect on the activation of GLI2, they are likely to block or impair the activity of the SHH signaling pathway, accounting for the pathogenesis of cleft lip/palate.

Recent studies have shown that the primary cilium, a microtubule‐based organelle that bulges out of the surface of quiescent cells, has a prominent role in modulating vertebrate SHH signaling (Eggenschwiler & Anderson, 2007). GLI2 has also been reported to translocate to and accumulate at the tips of primary cilia upon pathway activation (Chen et al., 2009; Kim, Kato, & Beachy, 2009; Wen et al., 2010). In addition, the levels of GLI2 protein are altered in many ciliary mutant mice, suggesting that the primary cilium is crucial for the normal function and proper control of GLI2 protein processing. However, whether the primary cilium plays a role in the pathogenesis of NSCL/P in this family and the nature of that role is still unknown. This study is merely a preliminary exploration of the potential causative mutations for the Chinese NSCL/P family. The exact pathogenic mechanisms of these three *GLI2* mutations leading to NSCL/P are not fully understood. Further demonstrations are needed through animal models and cellular/molecular experiments. Besides, the genetic contribution rates of these variants also remain to be explored in a large sporadic population.

In conclusion, we identified three candidate missense mutations in the coding region of *GLI2* by WES in a Chinese NSCL/P hereditary family. Preliminary studies such as functional prediction, conservation analysis, and homology modeling suggested that the variants c.2684C > T_p.Ala895Val, c.4350G > T_p.Gln1450His, and c.4622C > A_p.Ser1541Tyr potentially underlie NSCL/P in this pedigree. Our results further add to the increasing evidence that *GLI2* variants play a role in the pathogenesis of NSCL/P.

## CONFLICT OF INTEREST

The authors have declared no competing interests.

## AUTHORS’ CONTRIBUTIONS

P. Meng and H. Zhao conducted PCR and DNA cloning, analyzed data, and drafted the paper. W. Huang, Y. Zhang, and F. Chen analyzed data. W. Zhong, M. Zhang, and P. Jia collected human subjects. Z. Zhou and Gulibaha Maimaitili examined the affected patients. J. Zhang and J. Lin designed the study.
