# Supplementary material for: Three GLI2 mutations combined potentially underlie non‐syndromic cleft lip with or without cleft palate in a Chinese pedigree
Source: Mol Genet Genomic Med. 2019 Aug 6;7(9):e714. doi: 10.1002/mgg3.714 (PMC6732289; doi:10.1002/mgg3.714)
Supplement: Supplementary file 1 [file MGG3-7-e714-s001.pdf]

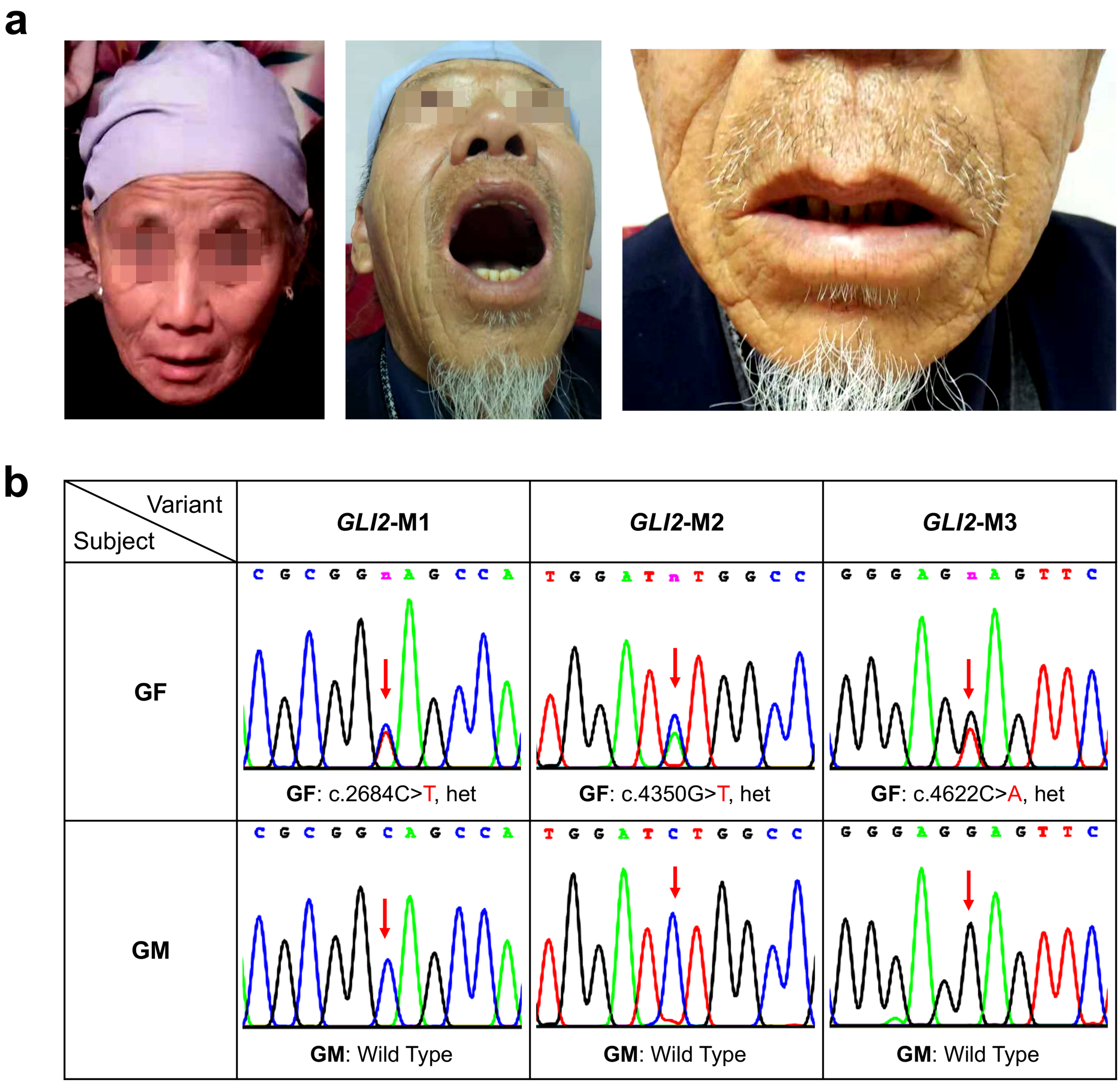

Supporting Information Figure S1 (a) Photographs of the parents of the subject D2. (b) Sanger sequencing of the three potential causative mutations in the parents of the subject D2 (GF: the father of the subject D2; GM: the mother of the subject D2)

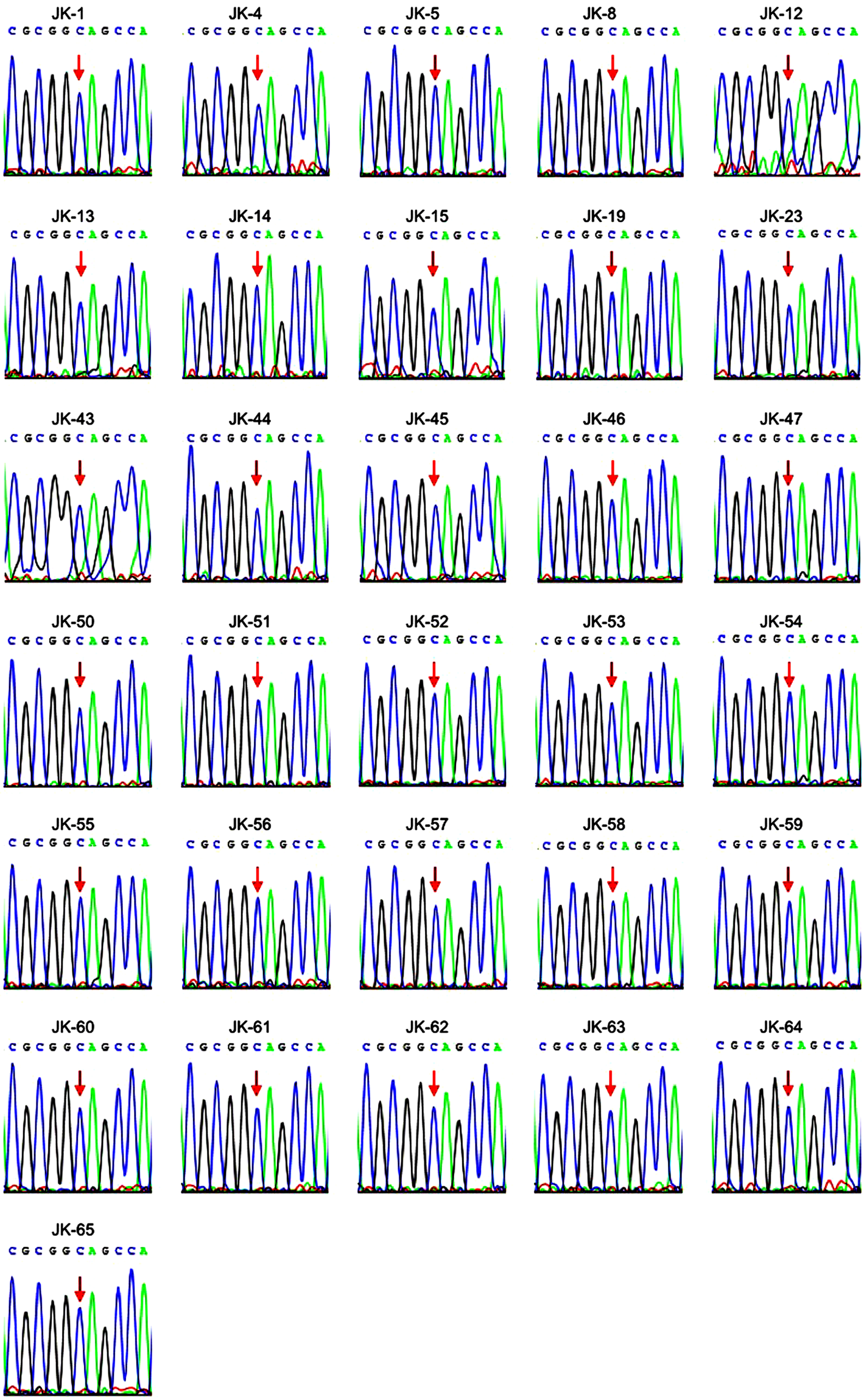

Supporting Information Figure S2 Sanger sequencing of *GLI2* mutation M1: c.2684C>T in 31 unaffected Chinese subjects

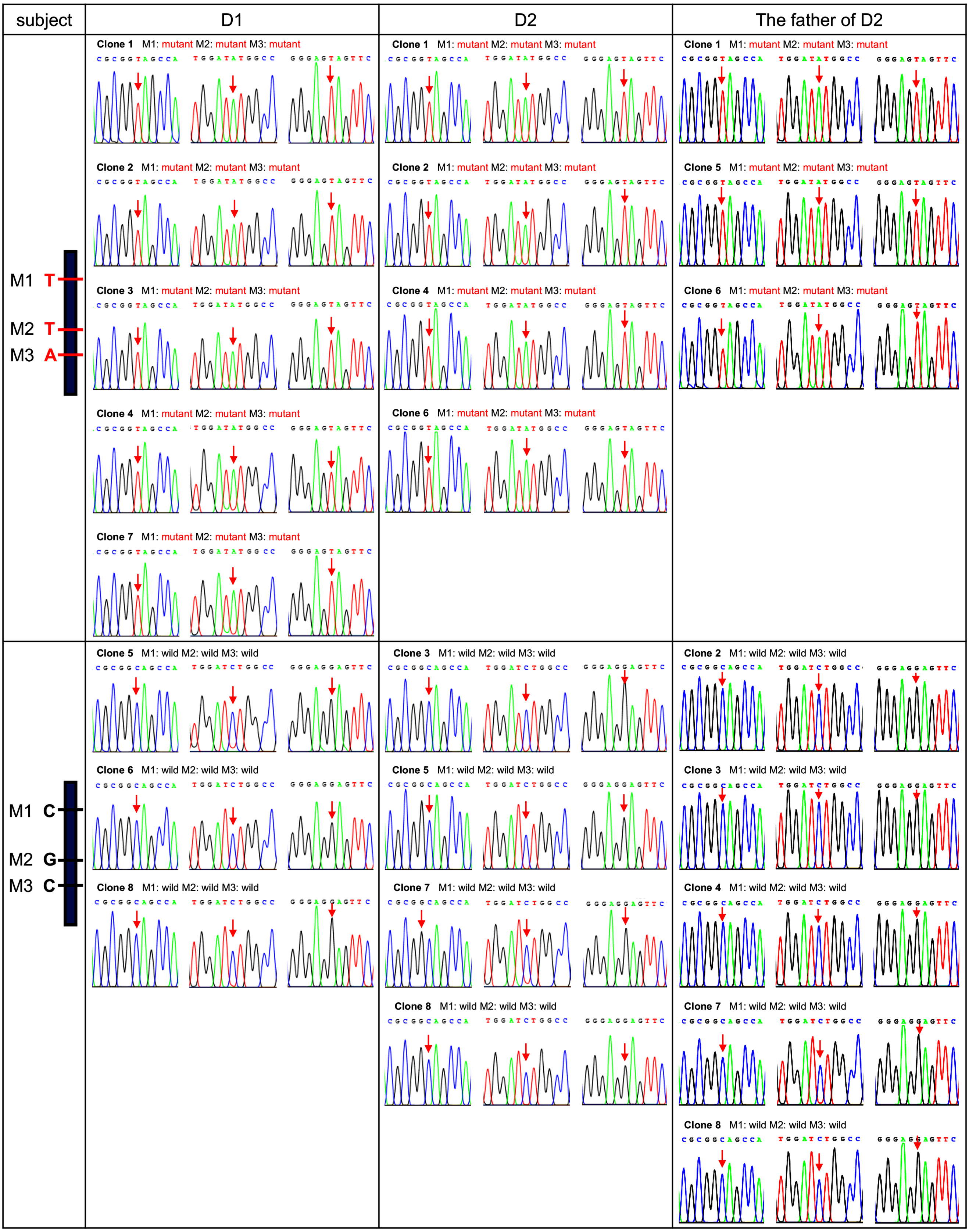

Supporting Information Figure S3 Sanger sequencing of eight clones containing DNA from subject D1, eight clones containing DNA from subject D2, and eight clones containing DNA from the father of D2

Supporting Information Table S1 Information of the fourteen candidate variants identified by whole-exome sequencing

No.	Gene	Mutation type	Predicted protein variants	SIFT	PROVEAN	PolyPhen2	MutationAssessor
**1**	*GLI2*	missense variant	NM_005270.4:c.4622C>A:p.Ser1541Tyr	0.01	-3.42	0.998	3.015
**2**	*GLI2*	missense variant	NM_005270.4:c.4350G>T:p.Gln1450His	0.00	-3.42	0.972	2.54
**3**	*GLI2*	missense variant	NM_005270.4:c.2684C>T:p.Ala895Val	0.02	-3.43	0.911	2.58
**4**	*C5orf42*	splicing region variant	NM_023073.3:c.3150T>A	-	-	-	-
**5**	*SLC19A1*	missense variant	NM_194255.2:c.61G>A:p.Glu21Lys	0.09	-1.402	0.775	1.21
**6**	*TCEB3*	missense variant	NM_003198.2:c.452G>A:p.Ser151Asn	0.00	-0.585	0.991	2.655
**7**	*WDR1*	missense variant	NM_017491.3:c.1568C>T:p.Ser523Leu	0.46	-0.214	0.103	2.15
**8**	*N4BP2*	missense variant	NM_018177.4:c.3227C>T:p.Thr1076Met	0.00	-2.713	1.000	2.095
**9**	*ITGB3*	missense variant	NM_000212.2:c.506G>A:p.Arg169Gln	0.33	-0.935	0.923	1.155
**10**	*ANK2*	missense variant	NM_001148.4:c.61G>A:p.Pro2560Ala	0.00	-1.794	0.995	1.655
**11**	*KISS1*	missense variant	NM_002256.3:c.107A>G:p.Gln36Arg	0.00	-1.809	0.100	0.975
**12**	*EMX1*	missense variant	NM_004097.2:c.263C>T:p.Pro88Leu	0.00	-2.339	0.968	1.905
**13**	*COL9A2*	missense variant	NM_001852.3:c.1753G>T:p.Val585Leu	0.60	-0.637	0.354	0.635
**14**	*ACE*	missense variant	NM_000789.3:c.2005G>A:p.Glu669Lys	0.12	-2.114	0.999	2.685
